# Identification of a ferroptosis-related long noncoding RNA signature with a prognostic value in adrenocortical carcinoma

**DOI:** 10.3389/fgene.2022.949457

**Published:** 2022-11-15

**Authors:** Weixi Wang, Guilin Chang, Ran Zhuo, Cong Ye

**Affiliations:** ^1^ Department of Geriatrics, Zhongshan Hospital, Fudan University, Shanghai, China; ^2^ Department of Urology, Zhongshan Hospital, Fudan University, Shanghai, China; ^3^ Department of Thoracic Surgery, Shanghai Pulmonary Hospital, Tongji University, Shanghai, China

**Keywords:** adrenocortical carcinoma (ACC), ferroptosis, lncRNA, immune infiltration, data mining

## Abstract

**Background:** Adrenocortical carcinoma (ACC) is an uncommon endocrine malignancy associated with poor clinical outcome. As a novel form of cell death, ferroptosis is reliant on the accumulation of iron and reactive oxygen species and is involved in the pathogenesis of various tumors, including ACC. Our study aimed to identify and characterize the prognostic ferroptosis-related lncRNA signature (FerRLSig) in ACC.

**Methods:** A regulatory network of ferroptosis-related lncRNAs (FerRLs) and mRNAs was constructed based on The Cancer Genome Atlas (TCGA). Univariate and multivariate Cox regression assays were performed to construct the FerRLSig.

**Results:** Twenty-four FerRLs were identified in the prognostic model, and the high-risk FerRLSig was related to the worse overall survival (OS) in ACC [hazard ratio (HR): 1.936 (1.484–2.526), *p* < 0.001]. The area under the curve (AUC) value of the FerRLSig was 0.936 according to the receiver operating characteristic (ROC) analyses, superior to other traditional clinicopathological features, further supported the utility in prognosis prediction of ACC. We further established a prognostic nomogram combining clinical factors with the FerRLSig, which showed favorable efficacy for survival prediction. Next, gene set enrichment analysis (GSEA) revealed that gene sets were involved in many immune regulatory biological processes related to malignancies. T-cell function of type II INF response and the immune checkpoints, including CD40, CD276, IDO2, NRP1, and CD80, were expressed with a significant difference between the low- and high-risk groups.

**Conclusion:** This study offered new insights into the pathogenesis of ACC. The novel FerRLSig could be useful in predicting survival and may provide information of immunological research and treatment for ACC patients.

## Introduction

Adrenocortical carcinoma (ACC) is an aggressive endocrine malignancy with a rare incidence but high mortality (Else et al., 2014; Zheng et al., 2016). Although patients could benefit from complete surgical resection or mitotane treatment, the prognosis of ACC is dismal with the 5-year survival of 20%–59% (Fassnacht et al., 2011; Zheng et al., 2016). In addition, the prognosis of ACC varies with the patient’s age, surgical scope, mitotic intensity, and secretion of hormones. Due to the tumor heterogeneity of ACC, the current tumor, lymph node, and metastasis (TNM) staging system is not reliable in clinical outcome prediction. It is challenging to make a prediction of the survival in ACC patients owing to the low incidence rate, diverse pathogenic determinants, high heterogeneity, and dismal prognosis. Therefore, it is particularly critical to uncover new and reliable prognostic biological markers for patients with ACC.

Past decades have seen a growing number of research studies to investigate the function of ferroptosis in malignancies. As a novel form of cell death, ferroptosis is reliant on the accumulation of intracellular iron and reactive oxygen species, distinguished from other forms of cell death such as apoptosis and autophagy (Dixon et al., 2012; Huang et al., 2020). Imbalance of iron metabolism could facilitate tumor growth and also act as a risk factor for tumorigenesis. Compared with normal cells, malignant cells excessively depend on iron for cell proliferation, that is, iron addiction (Manz et al., 2016). One recent study uncovered that ACC is remarkably sensitive to ferroptosis in human, and compared to mitotane, the ferroptosis inducer may be more specific, more effective, and less toxic for the treatment of ACC patients (Belavgeni et al., 2019). Weigand et al. (2020) demonstrated that adrenocortical cells are extraordinary sensitive to ferroptosis owing to the active steroid synthesis pathway. In addition, Chen and colleagues identified a ferroptosis-related signature which could be useful in prognosis prediction and immunotherapy or targeted therapy screening (Chen et al., 2021). Therefore, it is of vital importance to further investigate the changing profiles of ferroptosis-related genes and the underlying mechanisms in ACC.

As a subset of RNA molecules, a long noncoding RNA (lncRNA) has the length of over 200 nt and is involved in gene regulation (Zhang et al., 2021a). In addition, lncRNA is involved in various biological process and plays an important role in the development and progression of malignancies (Gupta et al., 2010), including ACC (Buishand et al., 2020). At present, few studies have focused on the functions of ferroptosis-related lncRNAs (FerRLs) in tumors. Recently, Chao et al. reported that cytosolic lncRNA P53RRA suppressed tumor progression by promoting ferroptosis. The P53RRA–G3BP1 interaction in the cytoplasm leads to the accumulation of p53 in the nucleus, conferring to ferroptosis and cell-cycle arrest. In patients with breast cancers or lung cancers harboring the wild-type p53, the decreased expression of P53RRA was significantly related to a poor prognosis (Mao et al., 2018), and p53 is considered as one of the most promising molecular targets for cancer therapy (Chasov et al., 2020). In another related study, Zhang et al. (2021b) demonstrated that the chronic cadmium exposure could significantly increase the expression of lncRNA OIP5-AS1 in prostate cancer. OIP5-AS1 regulates the expression of SLC7A11 (an alternative marker of ferroptosis) by sponging miR-128-3p, leading to enhanced ferroptosis and decreased cell viability. Similarly, LINC00336 serves as a competing endogenous sponge of miR6852 to inhibit ferroptosis in lung cancer (Wang et al., 2019a). However, no research has been performed to identify the function of FerRLs in predicting the overall survival (OS) for ACC patients. Thus, we constructed a prognostic ferroptosis-related lncRNAs signature (FerRLSig) according to The Cancer Genome Atlas (TCGA) database. Additionally, functions and biological pathways of ferroptosis-related genes and the N6-methyladenosine (m6A) mRNA status and immune checkpoint in the survival prediction of ACC patients were further explored.

## Materials and methods

### Data collection

On 5 February 2022, we extracted the RNA-sequence data of 92 patients from the TCGA-ACC data set (https://portal.gdc.cancer.gov/). Table 1 lists the clinical features, and the identified lncRNAs were annotated using the GTF annotation files of human lncRNAs retrieved from the GENCODE (https://www.gencodegenes.org/). We obtained 382 ferroptosis-related genes (*n* = 150 for the drivers, *n* = 109 for the suppressors, and *n* = 123 for the markers) from the FerrDb database, which is a data network with comprehensive and timely updates, covering the latest progress of ferroptosis-related genes along with their regulatory molecules and related diseases (Zhou and FerrDb, 2020) (http://www.zhounan.org/ferrdb, Supplementary Table S1). Information of the tumor grade in ACC was missing; thus, we collected clinicopathological data including gender, age, TNM stage, and survival information. Since the study was based on a public database, ethical approval is not required.

**TABLE 1:** Clinical characteristics of patients in TCGA dataset.

Variable	Number of samples
Gender	
Male/Female	32/60
Age at diagnosis	
≤65/>65	81/11
Stage	
I/II/III/IV/NA	9/44/19/18/2
T	
T1/T2/T3/T4/NA	9/49/11/21/2
M	
M0/M1/NA	69/21/2
N	
N0/N1/NA	80/10/2

### Identification of a prognostic FerRLSig

The relationship between the FerRLs and ferroptosis-related genes was assessed by Pearson correlation. Statistical significance was achieved at a correlation coefficient |*R* ^*2*^|>0.8 and *p* < 0.001. Next, a FerRLSig was constructed based on the univariate and multivariate Cox regression analyses. Information of age, gender, stage, and risk score was included in the multivariate Cox regression analysis. The risk score was calculated as the sum of (coefficient of lncRNA1 × lncRNA1 expression) + (coefficient of lncRNA2 × lncRNA2 expression) + + (coefficient of lncRNAn × lncRNAn expression). Taking the median value as the cut-off point, the lncRNAs were divided into two groups: high-risk group and low-risk group. Differentially expressed genes (DEGs) between the low- and high-risk groups were identified and statistically significant was considered at a false discovery rate (FDR) < 0.05 and |log_2_FC|≥1. A Kaplan–Meier survival analysis was then performed to evaluate the clinical correlation of risk stratification and patient prognosis. The FerRLSig was present as a risk plot, including the distribution of risk scores and survival status in ACC patients. We further calculated the area under the curve (AUC) value in the receiver operating characteristic (ROC) curve to evaluate the prediction accuracy of OS in ACC patients. Sensitivity and specificity of the FerRLSig were compared with other clinical and pathological factors *via* ROC curves and decision curve analysis (DCA) (Vickers and Elkin, 2006). Cytoscape 3.9.0 was used to visualize the co-expression network of ferroptosis-related genes, lncRNAs, and signaling pathways.

### Functional analyses of DEGs

Functions of DEGs were compared between the low-risk group and the high-risk group. We evaluated the associated biological functions using gene ontology (GO), including biological processes (BPs), molecular functions (MFs), and cellular components (CCs). Based on the data in the Kyoto Encyclopedia of Genes and Genomes (KEGG), we further analyzed the biological pathways using the ggplot2 package in R 4.1.2.

### Gene set enrichment analysis and the predictive nomogram

To explore potential molecular mechanisms of the FerRLSig, we performed a gene set enrichment analysis (GSEA) to define the enriched terms in the KEGG. Significant gene sets were achieved at *p*-value <0.05 (GSEA 4.2.2). Statistical significance was expressed by the normalized enrichment score (NES) and FDR (Subramanian et al., 2005). A nomogram was developed by integrating the FerRLSig and clinical characteristics to predict the 1-, 3-, and 5-year OS in ACC.

### Immune infiltration and gene expression analyses

Potential correlation of the FerRLSig and immune cells infiltration was analyzed using the CIBERSORT algorithms (Newman et al., 2015; Charoentong et al., 2017) and TIMER website (http://timer.comp-genomics.org/) (Li et al., 2017). The differences of immune responses between the two groups were illustrated on a cluster heatmap. Next, a single-sample gene set enrichment analysis (ssGSEA) was performed to analyze the scores of tumor-infiltrating immune cells along with their functions in TCGA. Furthermore, we retrieved the previous literature to extract potential immune checkpoints (Charoentong et al., 2017).

### Statistical analysis

R software (version 4.1.2), along with its appropriate packages, was used to perform the statistical analysis. The Wilcoxon test and unpaired Student’s t-test were used to analyze the non-normally and normally distributed variables, respectively. Differentially expressed genes between the low- and high-risk groups were identified using the Benjamini–Hochberg method. The association between FerRLs and clinicopathological characteristics of ACC patients was assessed by logistic regression analysis, presented in a cluster heatmap. Statistical significance was achieved at *p* < 0.05.

## Results

### Identification of a prognostic FerRLSig in adrenocortical carcinoma

According to the Pearson correlation analysis, a total of 85 FerRLs were identified from the 382 ferroptosis-related genes in TCGA-ACC dataset (Supplementary Table S2). Subsequently, 32 significant FerRLs were screened by the univariate analysis and then included in the multivariate Cox analysis. Overall, 24 FerRLs (AC046143.1, AC099850.3, LINC01614, LASTR, DPP4-DT, AL391422.4, LINC01503, LINC01843, AC008105.2, AC004816.2, AC112715.1, AC012073.1, LINC00460, U62317.1, BX470102.1, LINC02268, GAPLINC, MYOSLID, AC123912.4, AC091057.1, DGUOK-AS1, PRKAR1B-AS1, MIR222HG, and AL513477.2) were identified to be independent prognostic factors for ACC (Supplementary Table S3). Annotations and potential mechanisms of these identified FerRLs are presented in Supplementary Table S4. Next, a prognostic FerRLSig was constructed based on the calculated risk scores.

**FIGURE 1:**
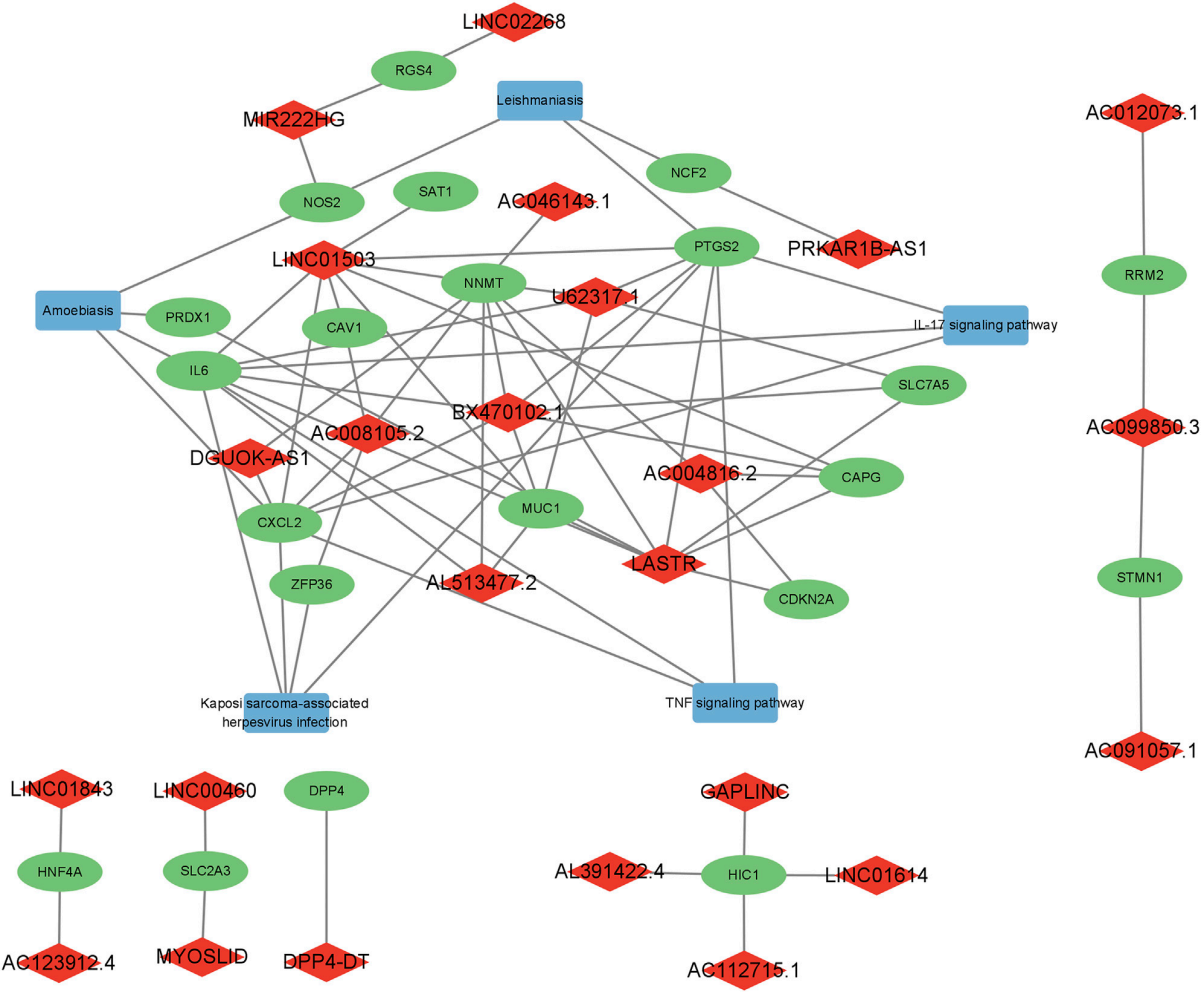
Regulatory network of the ferroptosis-related lncRNAs, mRNAs, and signaling pathways. Diamonds represent lncRNAs in red, rectangles indicate signaling pathways in blue, and ellipses represent mRNAs in green.

The regulatory network of FerRLs, mRNAs, and pathways is shown in Figure 1, which indicated that these FerRLs regulate multiple ferroptosis-related pathways by targeting the mRNAs. For example, lncRNA BX470102.1 is involved in amoebiasis, Kaposi sarcoma-associated herpesvirus infection, TNF signaling pathway, and IL-17 signaling pathway by targeting multiple target genes such as IL-6, CXCL2, and PTGS2. The target genes of LINC01503, U62317.1, AC008105.2, LASTR, and AC004816.2 are also involved in the aforementioned pathways, which have been proved to be related to ferroptosis in multiple tumors (Liu et al., 2021).

### Enrichment analysis of the identified ferroptosis-related genes

**FIGURE 2:**
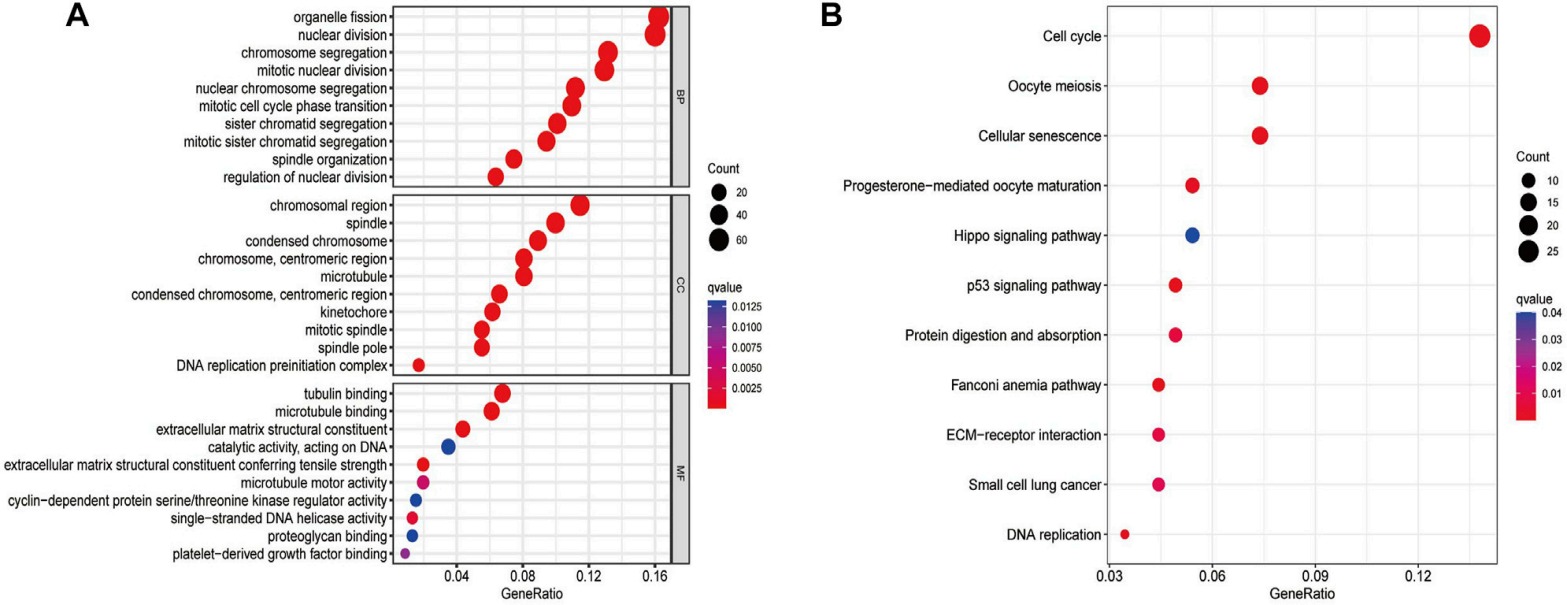
GO **(A)** and KEGG **(B)** analyses for ferroptosis-related genes differentially expressed between the low- and high-risk groups.

Overall, 540 genes were identified to be differentially expressed between the low- and high-risk groups (n = 103 for the low-risk group and *n* = 437 for the high-risk group, Supplementary Table S5). BP participated in organelle fission, mitotic nuclear division, chromosome segregation, and mitotic cell cycle phase transition. CC were mainly involved in the chromosomal region, mitotic spindle, and DNA replication preinitiation complex pathways. MF mainly regulated the single-stranded DNA helicase activity, cyclin-dependent protein serine/threonine kinase regulator activity, tubulin binding, and extracellular matrix structural constituent (Figure 2A). The KEGG pathway analyses revealed that the genes in the high-risk group were particularly involved in oocyte meiosis, cell cycle, DNA replication, protein digestion and absorption, Hippo signaling pathway, small-cell lung cancer, cellular senescence, and p53 signaling pathway (Figure 2B; Supplementary Table S6).

### Prognostic value of the FerRLSig

**FIGURE 3:**
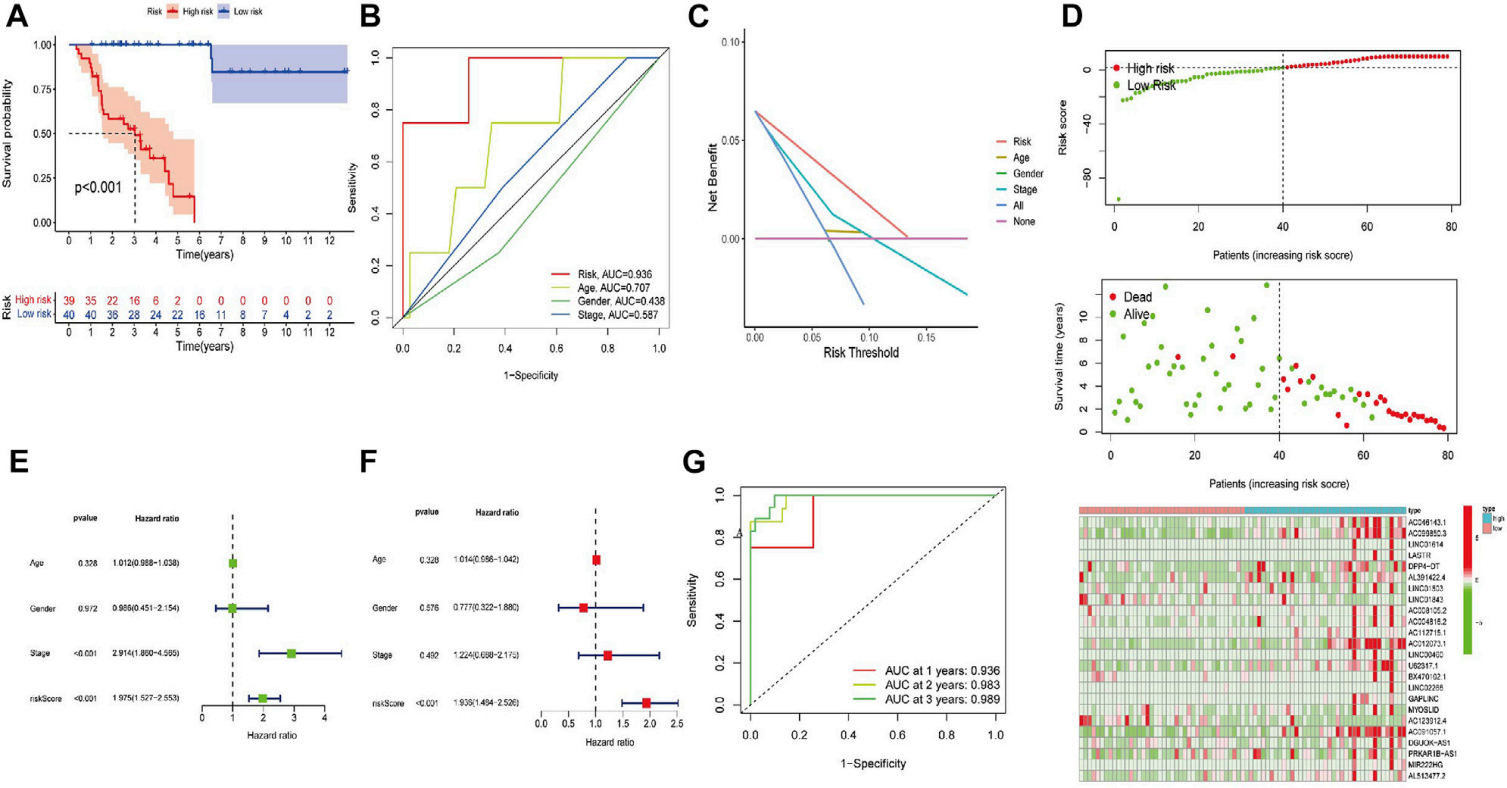
Construction of the ferroptosis-related lncRNA signature (FerRLSig). **(A)** Kaplan–Meier survival analysis stratified by the risk scores. **(B)** AUC values of the FerRLSig and other clinicopathological factors. **(C)** Decision curve analysis (DCA) of the risk factors. **(D)** Distribution of ACC patients based on the FerRLSig. Red and green dots represent the status of death and survival, respectively. The cluster heatmap represents the expressions of ferroptosis-related lncRNAs (FerRLs) stratified by the risk scores. **(E)** Univariate analysis of the risk score and other traditional clinicopathological factors. **(F)** Multivariate Cox analysis of the risk score and other traditional clinicopathological factors. **(G)** AUC value for the prediction of the 1-, 2-, and 3-year OS of ACC patients in the FerRLSig.

High-risk FerRLs were associated with a worse OS according to the Kaplan–Meier analyses (Figure 3A, *p* < 0.001). In addition, the AUC value for the FerRLSig was 0.936 according to the ROC curve, significantly higher than that for traditional clinicopathological factors such as gender, age, and tumor stage (Figures 3B,C), further supporting the potential superior performance of the FerRLSig in predicting the survival in ACC. As shown in the prognostic curve and scatter plot, the risk scores of ACC patients were negatively correlated with their OS. Meanwhile, the cluster heatmap revealed the elevated expressions of candidate FerRLs in the high-risk group (Figure 3D), which needs to be further explored.

**FIGURE 4:**
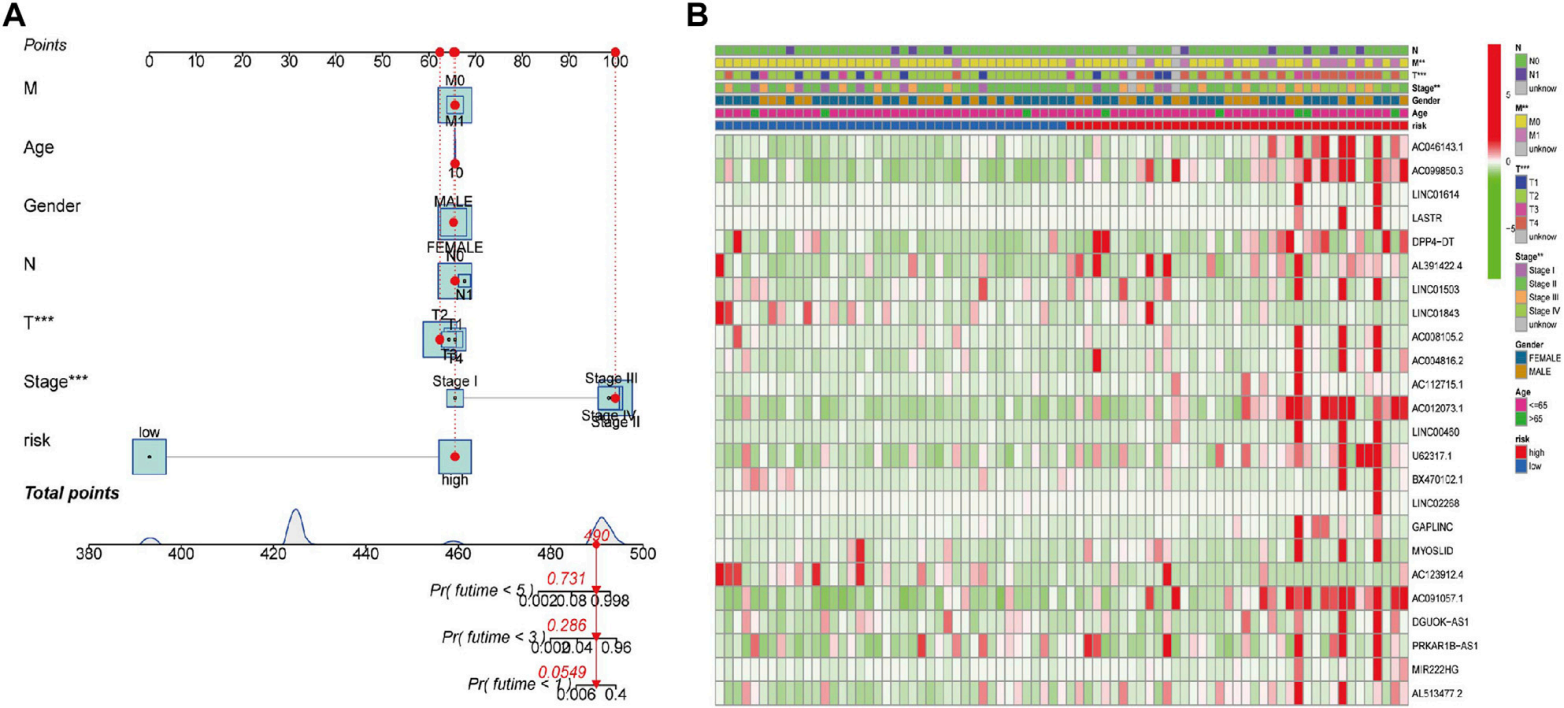
**(A)** A nomogram for the identified FerRLs and other clinicopathological factors. **(B)** Cluster heatmap for the association of clinicopathological manifestations and the prognostic FerRLSig.

To explore whether the generated FerRLSig could independently predict the OS for patients with ACC, we then performed univariate and multivariate Cox regression analyses. The hazard ratios (HRs) of the risk scores in the univariate analysis and multivariate Cox regression analysis were 1.975 (1.527–2.533) and 1.936 (1.484–2.526), respectively (*p* < 0.001, Figures 3E,F). Additionally, the predictive accuracy of this novel signature was evaluated by a ROC analysis, with the AUC values of 0.936, 0.983, and 0.989 for the 1-, 2-, and 3-year survival rates, respectively (Figure 2G). To make the model more applicable in clinical management, a nomogram incorporating the FerRLSig and clinicopathological features was constructed to predict the 1-,3-, and 5-year OS of ACC patients based on TCGA database (Figure 4A). The cluster heatmap for the association of clinicopathological manifestations and FerRLSig is illustrated in Figure 4B.

### Identification of biological pathways in the prognostic FerRLSig

**FIGURE 5:**
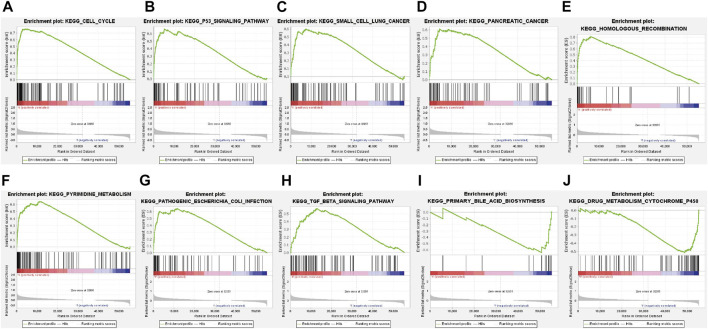
GSEA of the ferroptosis-related signaling pathways according to TCGA database. **(A)** Cell cycle, **(B)** p53 signaling pathway, **(C)** Small cell lung cancer, **(D)** Pancreatic cancer, **(E)** Homologous recombination, **(F)** Pyrimidine metabolism, **(G)** Pathogenic Escherichia coli infection, **(H)** TGF beta signaling pathway, **(I)** Primary bile acid biosynthesis, **(J)** Drug metabolism cytochrome P450 signaling pathway.

A total of 178 enriched KEGG pathways were identified in the GSEA (GSEA 4.2.2). Most of the identified FerRLs were involved in tumor-related and immunological pathways including cell cycle (NES = 2.07; *p* < 0.001), p53 signaling pathway (NES = 1.92; *p* < 0.001), small-cell lung cancer (NES = 1.75; *p* = 0.002), pancreatic cancer (NES = 1.74; *p* < 0.001), homologous recombination (NES = 1.91; *p* < 0.001), pyrimidine metabolism (NES = 1.89; *p* < 0.001), pathogenic *Escherichia coli* infection (NES = 1.78; *p* = 0.002), TGF-beta signaling pathway (NES = 1.67; *p* = 0.002), primary bile acid biosynthesis (NES = −1.66; *p* = 0.013), and drug metabolism cytochrome P450 (NES = −1.49; *p* = 0.027) signaling pathways (Figure 5 and Supplementary Table S7). Taken together, the novel prognostic signature may be involved in the microenvironment of tumor immune.

### Ferroptosis-related genes and immunity

**FIGURE 6:**
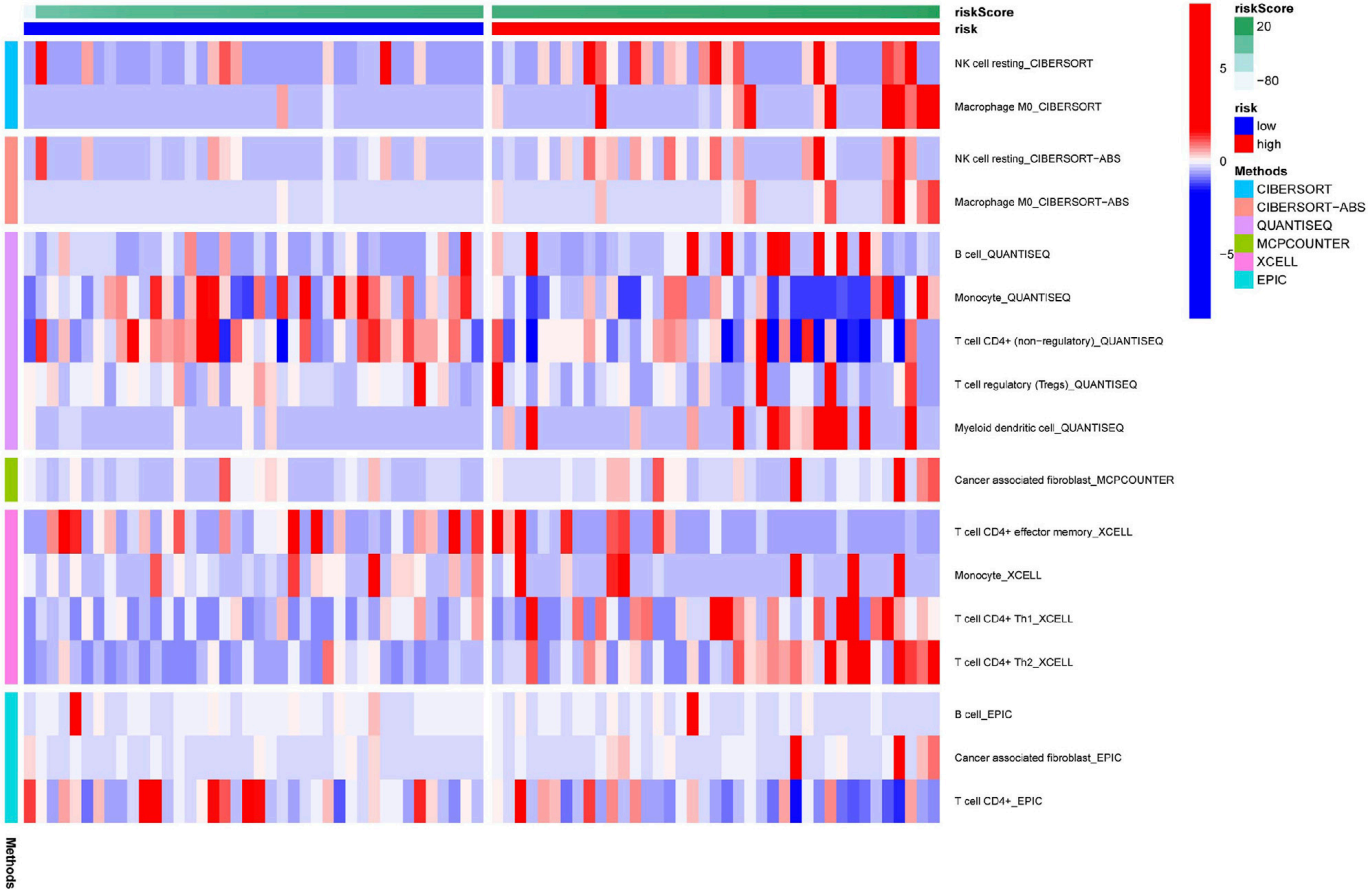
Immune response correlated to the ferroptosis-related lncRNA signature, illustrated in a cluster heatmap.

**FIGURE 7:**
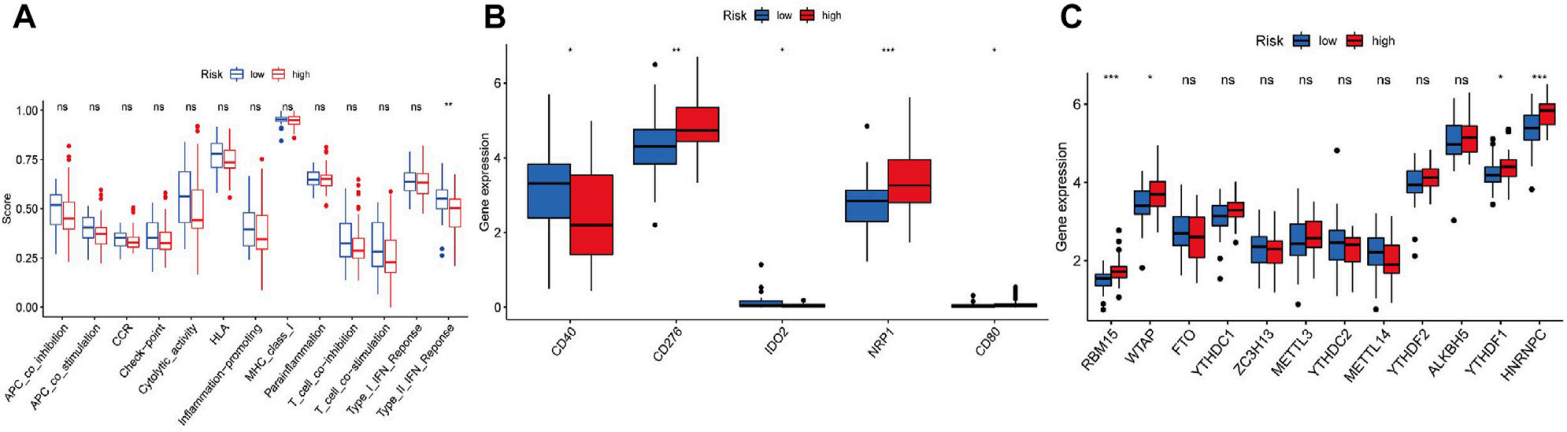
**(A)** Correlation analysis for immune cell subsets and the related functions by ssGSEA. **(B)** Comparisons of immune checkpoints expressions between the low- and high-risk groups. **(C)** Expressions of m6A-related genes based on the FerRLSig.

Figure 6 illustrates the immune responses clustering heatmap based on TIMER and CIBERSORT algorithms, which showed that immune infiltration was increased in cases with high-risk scores. Based on the ssGSEA of TCGA-ACC data, correlation analyses were performed to evaluate the association of immune cell subsets and corresponding functions, which revealed that T-cell function of type II INF response decreased significantly in the high-risk group (Figure 7A), suggesting that high-risk patients might be related to a suppressive immune microenvironment. Given the clinical importance of the checkpoint blocking therapy in tumors, we further analyzed the expression differences of immune checkpoints between the two groups, and the expressions of CD40, CD276, IDO2, NRP1, and CD80 were of significant differences (Figure 7B). The comparison of m6A-related mRNA expression between the two risk groups showed that RBM15, WTAP, YTHDF1, and HNRNPC were differentially expressed (Figure 7C). The aforementioned results indicate that the generated FerRLSig might be a candidate marker for the immune checkpoint inhibitor therapy in ACC patients.

## Discussion

ACC is an uncommon endocrine malignancy associated with high mortality (Wang et al., 2019b). Early diagnosis and more accurate therapies are of great importance to improve the prognosis. Some nomograms and risk models have been constructed to predict the survival in ACC (Wang et al., 2021a; Chen et al., 2021); however, no prognostic FerRLSig has been identified. Ferroptosis is associated with the development and progression of malignancies and might be a potentially novel approach for tumor therapy; additionally, its pivotal role in ACC has recently been revealed (Belavgeni et al., 2019). In this study, a FerRLSig that can evaluate the prognosis of ACC was constructed based on TCGA-ACC dataset. We further explored the association of survival and immune infiltrating cells and immune checkpoint inhibitors, which could provide potential biomarkers and immune targets for ACC treatment.

Overall, 24 FerRLs were identified in the prognostic FerRLSig. In review of the literature, cell types that predominantly express these FerRLs along with the potential mechanisms are presented in Supplementary Table S4. The AUC value of the ROC analysis indicated that the FerRLSig might have a superior predictive performance for OS in ACC. Additionally, a nomogram incorporating the FerRLSig and clinicopathological features of ACC was constructed to facilitate the development of treatment strategies and might guide clinical decision-making. One recent study revealed that overexpression of lncRNA AC099850.3 could promote cell proliferation and invasion *via* the PRR11/PI3K/AKT pathway, predicting a poor prognosis in hepatocellular carcinoma (Zhong et al., 2022). In a related study, LINC01614 was significantly upregulated in osteosarcoma and could facilitate tumor progression through the miR-520a-3p/SNX3 axis (Cai et al., 2021). In addition, the overexpression of LINC01614 in bladder cancer could promote tumor proliferation, migration, and invasion through the miR-217/RUNX2 and Wnt/β-Catenin pathway (Wang et al., 2021b). Upregulated expression of lncRNA LASTR was found in several epithelial tumors and could facilitate cancer cell fitness in hypoxic breast cancer through SART3 (De Troyer et al., 2020). LINC01503 is involved in a variety of malignancies, including hepatocellular carcinoma, cholangio-carcinoma, non-small-cell lung cancer, cervical cancer, and gastric cancer (Qu et al., 2019; Shen et al., 2020; Wang et al., 2020; Zhang et al., 2020; Feng et al., 2021; Ma et al., 2021). Wu et al. (2021) reported that the overexpression of LINC00460 in pancreatic ductal adenocarcinoma could promote tumor aggressiveness by targeting miR-491-5p, conferring a poor clinical outcome. Similarly, LINC00460 in head and neck squamous cell carcinoma facilitates peroxiredoxin-1 entry into the nucleus, thereby promoting cell proliferation and metastasis (Jiang et al., 2019). Knockdown of lncRNA GAPLINC repressed the tumor growth in renal cell cancer, while overexpression of GAPLINC facilitated tumorigenesis *via* the miR-135b-5p/CSF1 axis, indicating poor survival (Wang et al., 2021c). On the other hand, Yang and colleagues revealed that lncRNA MYOSLID played a critical role in the progression of osteosarcoma through the miR-1286/RAB13 axis (Yang et al., 2019a). In cervical cancer, upregulated expression of LncRNA DGUOK-AS1 could sponge miR-653-5p to repress DNA repair and promote cell proliferation (Wu et al., 2020). However, to the best of our knowledge, there has been no research conducted to explore whether FerRLs participate in the pathogenesis and prognosis of ACC.

Next, the present study identified 540 ferroptosis-related DEGs and stratified them by the novel FerRLSig. KEGG-based analyses further revealed that the genes of the high-risk group mainly participated in the Hippo signaling pathway, p53 signaling pathway, cancer-related pathway, ECM–receptor interaction, cellular senescence, and cell cycle. Recently, Lin and colleagues revealed that recurrent breast tumors are highly sensitive to ferroptosis, and upregulated expression of DDR2 could promote ferroptosis through the Hippo signaling pathway (Lin et al., 2021). Wen-Hsuan et al. reported that TAZ (a Hippo pathway effector) mediates the cell density-regulated ferroptosis by regulating the EMP1-NOX4 axis in renal cell carcinoma (Yang et al., 2019b). Another related study identified an ACSL4-independent and ALOX12-mediated pathway of ferroptosis that is essential for p53-dependent tumor suppression (Chu et al., 2019). Taken together, the aforementioned signaling pathways are closely related to ferroptosis in tumors.

Another significant contribution of the current study is the uncovering of the association between the FerRLSig and tumor immune microenvironment. Increasing evidence suggests that T-cells could promote tumor ferroptosis to enhance antitumor activity, which might be a potential therapeutic target for cancers combining with an immune checkpoint blockade (Wang et al., 2019c). Functional enrichment analyses revealed that FerRLs mainly participated in tumor-related and immune pathways. Type II INF response in T-cell function was significantly decreased in the high-risk group, suggesting that FerRLs may be involved in the regulation of tumor immune infiltration.

As a novel form of cell death, ferroptosis provides a new way for tumor development and treatment (Hassannia et al., 2019; Liang et al., 2019). However, the exact mechanism of ferroptosis in ACC remains unclear. To date, we are the first to construct a FerRLsig for ACC, which could shed light on novel treatment modalities for this rare tumor. Nonetheless, several limitations exist in the present study. First and most importantly, our findings are based on a single database and have not been validated by a clinical cohort. We explored the Gene Expression Omnibus (GEO) and the International Cancer Genome Consortium (ICGC); however, ACC is extremely rare and only 26 ACCs were found in the GEO, and no case of ACC was found in the ICGC. Thus, we could not compare or validate our findings with other data sets. In addition, the retrospective nature of this study made it less applicable. In addition, potential predictors such as tumor markers and tumor grade were not included in the research due to the incomplete data. Furthermore, functional experimental validation of the molecular mechanisms of the generated FerRLSig is currently lacking. In general, the role of the prognostic signature established in this study needs further exploration, and international multicenter studies with a larger sample size are expected.

In conclusion, a FerRLSig was established to predict the survival of ACC. Additionally, this model might be related to immune infiltration, providing potential immune targets for the control of ACC.

## Data Availability

The datasets presented in this study can be found in online repositories. The names of the repository/repositories and accession number(s) can be found in the article/Supplementary Material.
